# Characterizing the Internal Structure of Chinese Steamed Bread during Storage for Quality Evaluation Using X-ray Computer Tomography

**DOI:** 10.3390/s23218804

**Published:** 2023-10-29

**Authors:** Yonghui Yu, Chanchan Jia, Jiahua Wang, Fuwei Pi, Huang Dai, Xiaodan Liu

**Affiliations:** 1 College of Food Science and Engineering, Wuhan Polytechnic University, Wuhan 430023, China; yyh18371028765@163.com (Y.Y.); xibeinvdanchan@163.com (C.J.); w.jiahua@163.com (J.W.); huangdai9@126.com (H.D.); 2 Key Laboratory for Deep Processing of Major Grain and Oil (Wuhan Polytechnic University), Ministry of Education, Wuhan 430023, China; 3 School of Food Science and Technology, Jiangnan University, Wuxi 214000, China; pifuwei@jiangnan.edu.cn

**Keywords:** Chinese steamed bread, X-ray computer tomography, internal structure, quality evaluation

## Abstract

Chinese steamed bread (CSB) is a traditional food of the Chinese nation, and the preservation of its quality and freshness during storage is very important for its industrial production. Therefore, it is necessary to study the storage characteristics of CSB. Non-destructive CT technology was utilized to characterize and visualize the microstructure of CSB during storage, and also to further study of quality changes. Two-dimensional and three-dimensional images of CSBs were obtained through X-ray scanning and 3D reconstruction. Morphological parameters of the microstructure of CSBs were acquired based on CT image using image processing methods. Additionally, commonly used physicochemical indexes (hardness, flexibility, moisture content) for the quality evaluation of CSBs were analyzed. Moreover, a correlation analysis was conducted based on the three-dimensional morphological parameters and physicochemical indexes of CSBs. The results showed that three-dimensional morphological parameters of CSBs were negatively correlated with moisture content (Pearson correlation coefficient range−0.86~−0.97) and positively correlated with hardness (Pearson correlation coefficient range−0.87~0.99). The results indicate the inspiring capability of CT in the storage quality evaluation of CSB, providing a potential analytical method for the detection of quality and freshness in the industrial production of CSB.

## 1. Introduction

Chinese steamed bread (CSB) is a traditional staple food in China, popular with consumers due to its soft and delicious taste, high nutritional value, and affordable price [1]. At present, the demand for CSB is greatly increasing, with an annual consumption of more than 21 million tons. It is very important to realize the industrial production of CSB in order to meet market demands. However, the storage quality and freshness of CSB has always been a key problem that restricts the development of the CSB industry [2]. Specifically, the aging of CSB, caused by water migration and structural changes during storage, is an important factor that affects the flavor and taste [3]. Moreover, spoilage of fresh CSB due to high moisture content during storage can also affect its sensory quality and safety [4,5]. Therefore, the study of the quality changes that occur in CSB during storage is vital for its safe and effective storage, providing further reference for the shelf life and industrial production of CSB.

Moisture content and texture indexes are critical parameters in the quality evaluation of CSB [6]. They are mainly determined through physical and chemical methods, which are accurate and reliable but are also time-consuming and require destructive pretreatment [7,8]. Previous studies have shown that the microstructure of CSB is closely related to texture and moisture changes, reflecting its quality [9,10]. Thus, it is of great significance to study the internal structure of CSB. X-ray computer tomography (CT) is a nondestructive three-dimensional imaging and detection technology used for characterizing the internal structure of various materials [11,12]. The principle is that an accurately collimated X-ray beam and a highly sensitive detector are used to scan the cross-section of the detected object, generating two-dimensional (2D) slice images from different angle projections [13]. Then, three-dimensional (3D) volume is obtained by superimposing the sequence of 2D slice images [14,15]. 

With the advantages of fast detection speed, clear imaging, non-destruction and lack of pretreatment, CT techniques have been successfully applied in the internal quality evaluation of foods and agricultural products [16,17] in recent years. Donis-Gonzalez et al. used CT images for the noninvasive postharvest classification of coarse-fiber asparagus, reaching a classification accuracy of 91.2% based on the presence of tough fibrous tissues in asparagus, The results showed the great potential of CT in predicting asparagus quality [18]. Yanxin Duan et al. applied X-ray computed microtomography (μCT) techniques to study pine spot disease in the famous pear variety “Chili” in China, showing that the internal organization, size, and location of cork spots in pear fruits could be clearly and accurately identified [19]. Bhupendra M Ghodki et al. studied the effect of bread formulation on bread microstructure and crumb using μCT combined with image analysis [20]. At the same time, X-ray CT images can be combined with various algorithms, such as machine learning [21] and convolutional neural networks [22], to improve the accuracy of the structure. To the best of our knowledge, the characterization of the internal structure of CSB during storage based on X-ray CT has not been investigated. 

In this study, we aim to explore the feasibility of X-ray CT in evaluating the quality changes in CSB during storage. We characterized and visualized the internal structure of CSB during storage using the X-ray CT technique. Combined with image processing methods, characteristic parameters reflecting the internal microstructure of CSBs were obtained. Additionally, physicochemical indexes reflecting the quality of CSBs were attained through conventional detection methods. Moreover, we conducted Pearson correlation analyses of CT characteristic parameters and physical and chemical indexes to explore the relationship between the internal microstructure and storage quality of CSB, which could provide a theoretical basis and a potential tool for evaluating the storage quality of food like CSB.

## 2. Materials and Methods

### 2.1. CSB Samples

**Figure 1:**
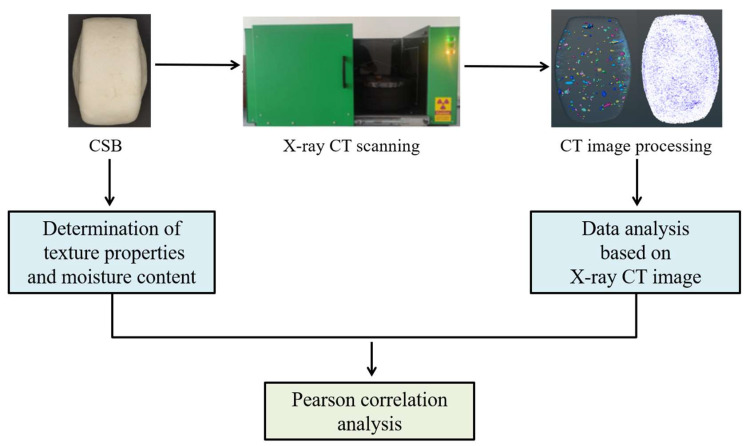
Flowchart of experimental methods.

CSB samples were purchased from the canteen of Wuhan Polytechnic University. They were then stored at room temperature until they became moldy after five days of storage. The average temperature was 24.7 °C, and the humidity was 44~55%. Three samples were used for the determination of physicochemical indexes (hardness, flexibility, and moisture content) and microstructure each day. The average values of physicochemical indexes were taken as the texture parameter and moisture content for each storage period, and the average values of parameters based on CT images were used for analysis. In order to intuitively reflect the microstructure changes of CSB during storage, the other three samples were applied to X-ray CT image scanning for 5 consecutive days. The analysis flowchart is presented in Figure 1, using a total of 18 samples.

### 2.2. Determination of Texture Properties

In order to ensure that the samples were flat and uniformly stressed to reduce experimental errors, CSB samples were cut into 4 uniform slices, with thicknesses of around 15 mm along the length direction [23], and 2 slices at the center of the sample were measured using a physical property analyzer (TMS-Pro; Beijing, China). The texture profile analysis (TPA) mode was adopted, with a probe of 25.4 mm, with the following test parameters: 30 mm rebound height, 180 mm/min test speed, 50% compression ratio, and 1 N minimum trigger force.

### 2.3. Determination of Moisture Content

In order to reduce the interference of external conditions, 4 uniform slices were then taken as samples, each with a thickness of 1 cm at the top and bottom of CSBs in the height direction were removed [23]. The middle part was then divided into three parts for follow-up experiments, including skin, crumb (the part 1 cm away from the skin), and center (the remaining part after removing the skin and crumb). The moisture content of each part of the CSB was determined according to the approved method 44-15A (AACC, 2000) [24].

### 2.4. X-ray CT Image Acquisition and Analysis

A desktop X-ray CT scanner (model NAOMI-CT 002L; RF Co., Nagano, Japan) at an accelerating voltage of 50 kV and a current of 10 μA was applied for the microstructure analysis of CSB. Individual samples were placed in the center of the turntable and X-ray CT images were obtained with a voxel size of 160 μm (spatial resolution) in normal mode and saved in RAW format. The scanning parameters used in the normal mode are listed as follows: imaging area of Φ146 × 153 − 185 mm, voxel number of 900 × 900 × 1100, grayscale values ranging from −32,768 to 32,767 (16 bit), and scan time of 120 s. The imaging area refers to the area covered during CT scanning, where “Φ146” indicates the diameter of the scanning area and “153–185 mm” indicates the height range of the scanning area. This was optimized according to the size of the sample. After cropping and scanning, approximately 800 original CT image slices were obtained for each sample in the longitudinal direction (xy plane).

After the scanning, calculations and visual reconstructions of CSBs were conducted based on grayscale values using Avizo image processing software (version 9.0.1, Thermo Fisher Scientific, Waltham, MA, USA). Grayscale values are closely related to the degree of X-ray attenuation through the object, reflecting the density of the object. Generally, the greater the density of the object, the stronger the attenuation, leading to a higher grayscale value (GSV). Balanced histogram thresholding, a histogram-based thresholding method, was used to segment X-ray CT images of CSB. The optimal threshold value was selected based on the CT image, and voxels with GSVs below or above this threshold were referred to as background or foreground, respectively. For each CT image, the voxels in the foreground were defined as regions of interest (ROIs) using the threshold segmentation method. Subsequently, these ROI regions were digitally isolated, allowing for the visualization of porous structures, as well as the analysis of 3D geometric models and morphological parameters of microstructures, such as total porosity, cell volume size, and surface area of the samples [25,26]. The morphological parameters are summarized in Table 1.

## 3. Results and Discussion

### 3.1. Analysis of Physicochemical Indexes

Hardness and flexibility are the main parameters used to characterize the textural properties of CSBs [27,28], and can reflect CSB aging [29], the most common problem during storage. Additionally, moisture transfer and the loss of CSB during storage are important factors that affect its aging. Therefore, changes in moisture content can be used to evaluate the quality of CSB during storage [30].

**Figure 2:**
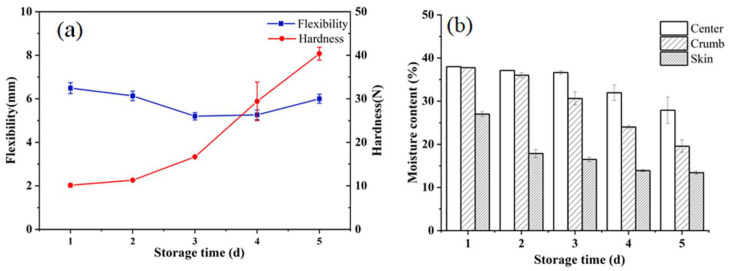
(**a**) Changes in TPA parameters (hardness, flexibility) and (**b**) moisture content of different parts (center, crumb and skin) of Chinese steamed bread (CSB) during storage (1d, 2d, 3d, 4d and 5d).

Figure 2a shows the hardness and flexibility of CSB during storage. In the TPA mode, hardness is defined as the sample’s maximum force during the first compression cycle [31]. It is obvious that the hardness of CSB gradually increased, from an initial 10.1 N to 40.4 N, which was consistent with the results of previous studies [32]. Furthermore, the flexibility of CSB showed an overall decreasing trend alongside the extension of storage time, from an initial 6.5 mm to 6.0 mm, which was similar to the results of previous studies [33]. The overall results indicate the aging of CSB during storage, mainly characterized by increased hardness and decreased flexibility.

Figure 2b shows the moisture content of the center, crumb, and skin of CSB during storage. It can be seen that the moisture content of these three parts of CSB showed a decreasing trend, as a whole. In addition, the moisture content of each part of CSB was different, with the skin having the lowest moisture content while the center had the highest. Specifically, in the early stages of storage, the moisture content in the center and crumb of CSB remained basically unchanged, while the moisture content of the skin decreased rapidly, which might be credited to the large contact area between the CSB skin and the air [34]. After 1 day of storage, the moisture content in the crumb and center parts of CSB began to decrease, with the moisture content of the crumb decreasing faster than that of the center. Meanwhile, the moisture content in the CSB skin decreased more slowly than before, likely due to moisture migration from the center to the skin caused by differences in water concentration [35]. Moreover, during the storage of CSB, the increase in hardness corresponded to moisture migration and loss, which was consistent with previous research [27].

### 3.2. Analysis of Internal Microstructure

#### 3.2.1. Images and Characteristics of CSB

**Figure 3:**
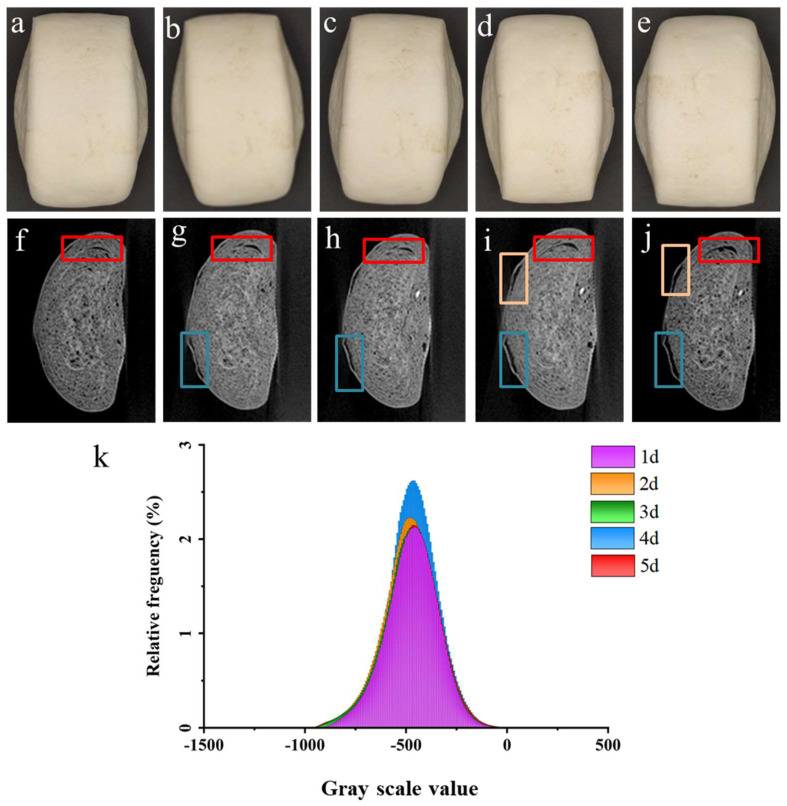
(**a**–**e**) Visual images, (**f**–**j**) X-ray CT images and (**k**) GSV histograms of CSBs during storage (1d, 2d, 3d, 4d and 5d).

Figure 3a–e shows visual images of CSB for 5 consecutive days, and no significant changes in the appearance of CSB during storage were seen. Figure 3f–j shows X-ray CT images of the same steamed bun, providing a better reflection of the internal information of CSB. It is clear in Figure 3f that the internal structure of fresh CSB was spongy with uniform, dense pores and a clear texture as a whole. There was also a phenomenon of delamination, as shown in the red box, which may be related to the preparation method of CSB [36]. Furthermore, the internal structure of CSB changed significantly with the extension of storage time, as seen in Figure 3f–j. Specifically, delamination of CSB was more obvious, and a distinct separation between the skin and crumb of CSB was observed in the blue and yellow boxes in Figure 3g–j and Figure 3i,j, forming a large hollow structure, which became more obvious with the increase in storage time. This phenomenon may be due to different rates of moisture loss between the skin and crumb of CSB during storage [37].

Figure 3h shows GSVs of CSB during storage. It is obvious that the GSVs of CSB were approximately between −750 to −250. Furthermore, the GSVs were basically constant during the whole storage period, which was consistent with existing studies [38]. Subsequently, the optimal threshold segmentation range (−750~−250) was determined to segment the internal pore structure from CSB. Parts with GSVs not in the above range represented air and pore structure. In addition, the pore structures inside CSB during storage were further analyzed based on the GSV tomographic images.

In order to further explore the microstructure changes of CSB during storage, individual pores in CSBs were measured using CT images and image processing software. Average values of morphometric parameters, including volume, surface area, length, and width, were calculated. Moreover, the average volume of the whole CSB was also calculated. The results are presented in Table 2.

It was noted that the volume of CSB gradually decreased during storage, which can be credited to the contraction of the cell wall [39], owing to the loss of water. As for individual pores, the volume and surface area gradually increased with the extension of storage time, which may be explained by two reasons. Firstly, the pores containing water were filled with air water was lost [40]. Secondly, the delamination in CSBs was more obvious during storage, due to water loss, as shown in Figure 3, forming larger holes, which in turn affected the volume and surface area of the pores. Additionally, the length and width of the pores also showed an upward trend during storage, increasing from the initial 3.25 mm to 3.98 mm and 1.65 mm to 1.71 mm, respectively, which was consistent with the changes in volume and surface area. Among them, the volume increased from 7.89 mm^3^ to 18.10 mm^3^, and the surface area increased from 19.53 mm^2^ to 35.31 mm^2^. In addition, the increases in pore morphometric parameters and the decrease in the volume of CSB together determined the change in porosity, which significantly increased. Especially in the later stages of storage, porosity changed from 23.5% to 28.8%. During ambient storage, the change in porosity was significant after 3 days, providing reference for the storage of CSB.

#### 3.2.2. Imaging of Pores and Pore Throat Distribution

**Figure 4:**
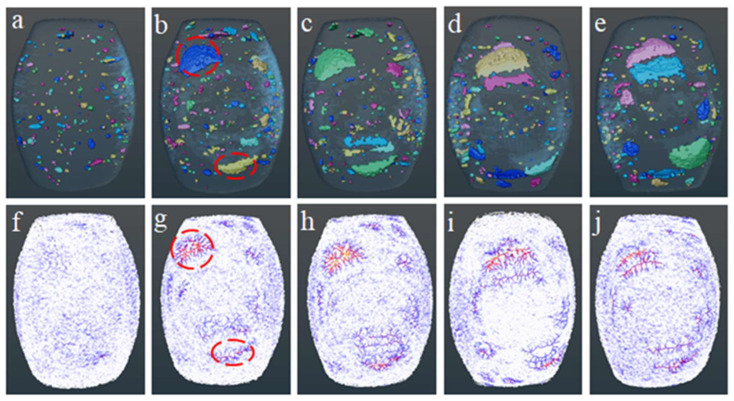
CSBs store pore distribution (**a**–**e**) and pore throat distribution (**f**–**j**) for 5 consecutive days (1d, 2d, 3d, 4d, and 5d).

Images in Figure 3, obtained from X-ray CT, were reconstructed in three dimensions and then subjected to different image processing techniques in order to obtain more concise and intuitive pictures of the internal microstructure in CSB. Specifically, the pores inside CSBs were separated from the reconstructed 3D image using the “Interactive Thresholding” method, and the image of the whole CSB was obtained using the “Fill Holes” method. Then, the image of all pores inside CSB was obtained using a simple “Arithmetic” method. Subsequently, the pores inside CSBs were separated using the “Opening” method, in which the “Ball” mode was used with a size larger than 3 voxels to obtain a clear image. Finally, the separated pores were subjected to “Label Analysis” to obtain the distribution of pores in CSB, as shown in Figure 4a–e. The image of all pores inside CSB, obtained using the “Arithmetic” method, was then processed by the “Auto Skeleton” method to obtain the pore throat distribution in CSB. A pore network model was used to perform a skeleton analysis. The distance image map was initially segmented, and the network model was then refined. Connection voxels were retained, and the voxel skeleton was converted into spatial graphic objects. The red filamentary structures in Figure 4g–j represent pore throats, which also reflect the connectivity between pores. The larger the pore throat radius, the better the connectivity between pores. In addition, the circles of the same size in Figure 4b,g are both in the same position in CSB.

As seen in Figure 4a–e, pore size and distribution of fresh CSBs were uniform, but these have obviously changed during storage. With the extension of storage time, more and more large pores appeared which might be due to water dissipation, cell wall contraction, and destruction inside the bun. Small pores were connected to each other to form larger ones [41], showing better connectivity between pores. Thus, the average volume and diameter of pores, as seen in Table 2, gradually increased during storage, eventually leading to an increase in porosity. In addition, it is obvious in Figure 4f,g that red pore throat structures gradually increased, gradually expanding its range, indicating a gradual increase in connectivity between the internal pores of CSB during storage. This trend was consistent with those seen in the internal structure of CSB in Figure 4a–e above.

**Figure 5:**
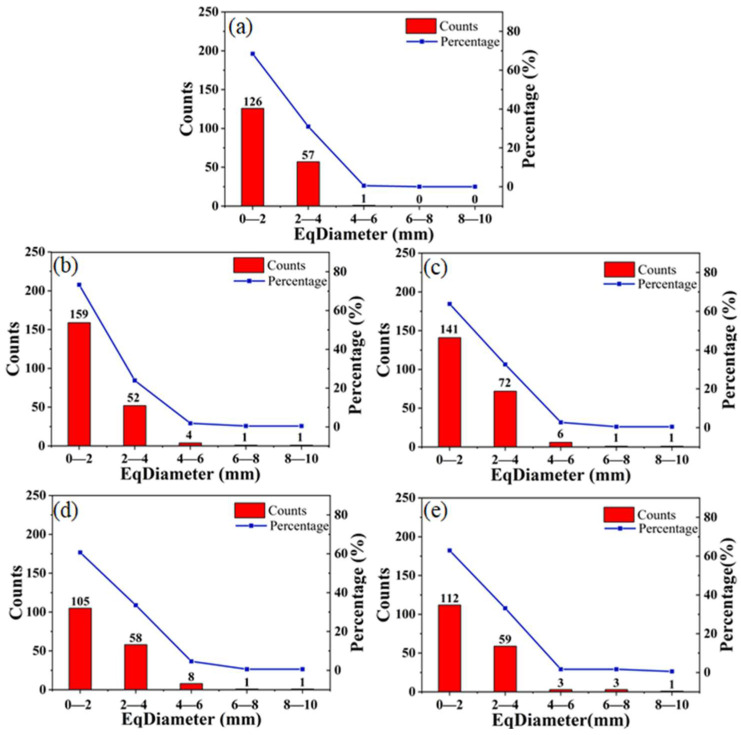
Pore size distribution (including counts and percentage variation) of CSB (**a**–**e**) stored continuously for 5 days (1d, 2d, 3d, 4d and 5d).

In order to quantitatively study the evolution of pores in CSB, the number and proportion of pores with different apertures in CSBs were calculated based on the reconstructed 3D image (Figure 5). For samples with porous structures, the shape of the internal pore structures were complex. Thus, “EqDiameter”, usually used for non-spherical structure, was introduced as the diameter of the pore.

As depicted in Figure 5, a large fraction of pores were observed in the diameter ranges of 0–2 mm and 2–4 mm. Specifically, at the initial stage (as shown in Figure 5a), no pores were observed in the diameter ranges of 4–6 mm, 6–8 mm, and 8–10 mm. During the whole storage process, the number of small pores showed an overall downward trend, while the number of macropores slightly increased. This phenomenon shows that macropores gradually appeared and small pores gradually disappeared during storage, which is consistent with Figure 4a–e above. The number of macropores with diameters greater than 6 mm in the aged CSB increased slightly, which might be due to the delamination caused by the loss of water in CSB.

#### 3.2.3. Analysis of Local Voxel Grayscale Values

**Figure 6:**
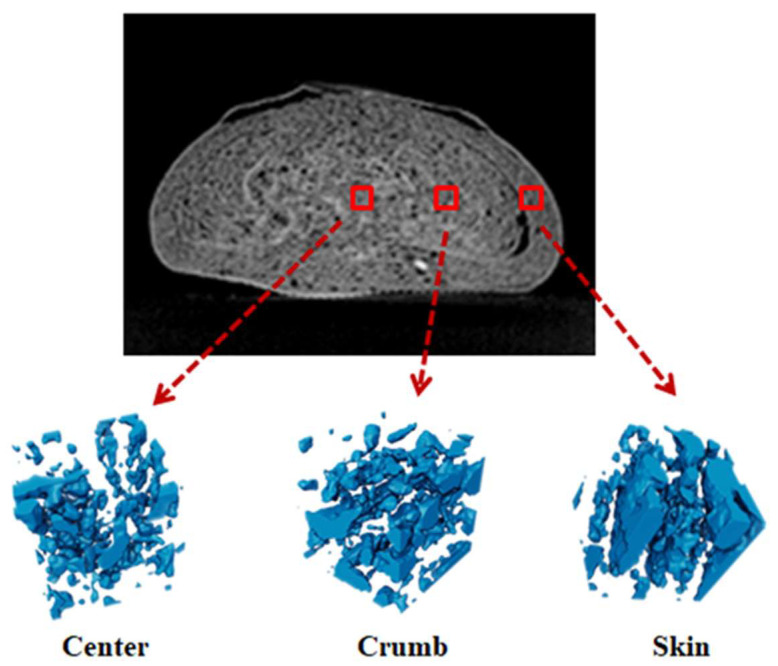
Internal microstructure of different parts of CSB based on 3D reconstruction of local ROI.

Local voxel grayscale value analysis was applied to further investigate the structural differences of the three parts of CSB. Three cubic regions with 45 voxels on each side were selected from three different parts (center, crumb, and skin) from the same horizontal position of the CSB. The ROI for reconstruction was still the same as that of the whole CSB. However, the segmentation threshold range of −1250~−1000 was selected, which reflected the pore structure in the CSB. Figure 6 shows the differences in the internal structure of the same CSB. The center has less pores and a tighter structure, while the skin has more pores. This may be related to the moisture content and moisture transfer in the CSB from the center to the skin during storage. In the process of moisture migration, moisture can form hydrogen bonds with more molecules, resulting in a tighter structure [42,43]. On the other hand, moisture can be used as a plasticizer to increase the flexibility and plasticity of the dough, so a higher moisture content in the CSB yields a finer texture.

### 3.3. Correlation Analysis

**Figure 7:**
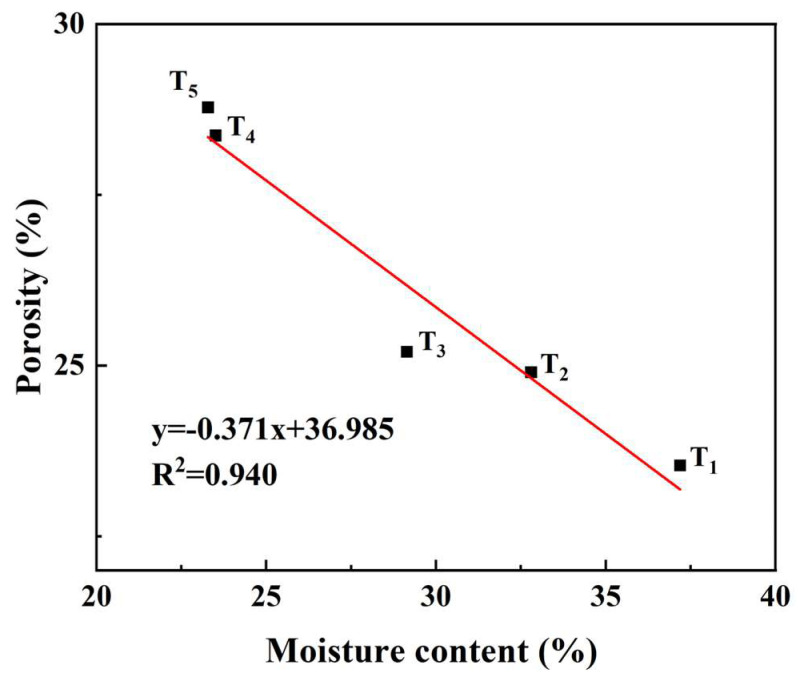
Linear fitting image of moisture content and porosity of CSBs stored for 5 days (1d, 2d, 3d, 4d, and 5d).

In order to further quantitatively study the relationship between the microstructure and moisture content of CSB, according to the results of the analysis of local voxel grayscale values, the moisture content and porosity of the steamed buns were initially analyzed. The above results show that the porosity gradually decreased with the increase in moisture content. Thus, the moisture content and porosity of the CSBs were linearly fitted. The results are shown in Figure 7, demonstrating that the moisture content of CSBs had a strong negative correlation with porosity, with a determination coefficient (R^2^) of 0.94.

**Figure 8:**
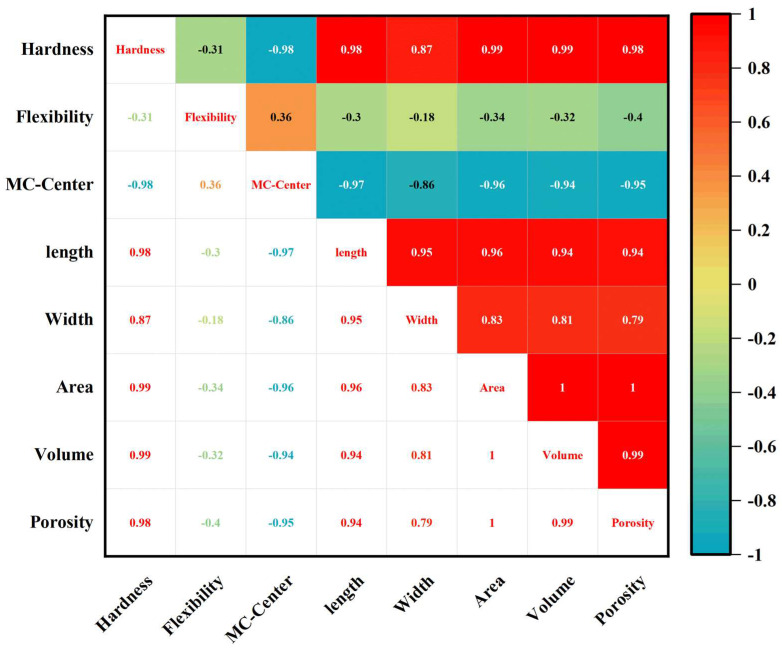
Pearson correlation matrix of physicochemical indicators and 3D morphological parameters of CSB.

In order to explore the correlation between other physicochemical indexes of the CSB and three-dimensional morphological parameters, all the parameters measured in this experiment were subsequently used for correlation analysis. Physicochemical indexes (hardness, flexibility, moisture content) and three-dimensional morphological parameters (volume, area, porosity, length, width) of the CSBs were analyzed using Pearson correlation coefficients. The results are shown in Figure 8.

As seen in Figure 8, the flexibility of CSBs were poorly correlated with other physicochemical indexes (notably hardness and moisture content), with Pearson correlation coefficients of −0.31 and 0.36, respectively. In addition, all three-dimensional morphological parameters of CSBs were positively correlated (0.79~1). Among them, the volume of these three-dimensional morphological parameters in CSBs were strongly correlated with the surface area, with a Pearson correlation coefficient of 1, due to strong correlations between changes in object volume and changes in surface area. More importantly, the moisture content of CSB was negatively correlated with hardness, with a Pearson correlation coefficient was −0.98. The moisture content of CSB was also negatively correlated with three-dimensional morphological parameters, with the maximum Pearson correlation coefficient reaching −0.97, while hardness was positively correlated with these three-dimensional morphological parameters. The results further confirmed that water loss affects the microstructure of CSB, thus leading to its hardening and aging. Therefore, it is feasible to evaluate CSB aging using microstructures based on CT images, combined with physicochemical indexes of CSB.

Compared to recent applications of X-ray CT in bread [20,44], a linear relationship between porosity based on CT images and moisture content, was established, which provides a potential method for the study of storage quality of foods like CSBs.

## 4. Conclusions

Non-destructive CT technology was applied to characterize and visualize the microstructure of CSB during storage, and physicochemical indexes were also analyzed. In addition, Pearson correlation analysis was conducted based on these physicochemical indexes and three-dimensional morphological parameters. The results showed that the hardness of CSB increased while the flexibility and moisture content decreased during storage. The moisture content of each part of CSB differed (center > crumb > skin), which could also be characterized by local voxel grayscale values based on CT images. Furthermore, the porosity of CSB increased significantly during storage, mainly related to the increase in the number of macropores caused by the loss of water in CSB. Moreover, three-dimensional morphological parameters of CSBs were negatively correlated with moisture content and positively correlated with hardness. In summary, X-ray CT was shown to be a powerful system for non-destructively and accurately mapping the internal microstructure of CSB, offering the potential for evaluating the storage quality of samples with porous structures, like CSB.

## Figures and Tables

**Table 1:** Morphological parameters for microstructure quantification of CSB.

Parameters	Unit	Function Description
Volume	mm^3^	The 3D volume of the object
Area	mm^2^	The area of the object boundary
Porosity	%	Total elements of the pore divided by the percentage of total elements of the analyzed sample
Length	mm	Maximum diameter of the analyzed object measured in one angle range
Width	mm	Minimum diameter of the analyzed object measured within a range of angles

**Table 2:** Changes of three-dimensional morphometric parameters during storage of CSB.

Storage Time/d	Volume (mm^3^)	Area(mm^2^)	Porosity (%)	Length (mm)	Width (mm)	Sample Volume (mL)
1	7.89 ± 2.60	19.53 ± 4.68	23.5 ± 2.78	3.25 ± 0.36	1.65 ± 0.07	165.33 ± 7.09
2	9.73 ± 3.27	22.04 ± 5.01	24.9 ± 2.88	3.16 ± 0.26	1.60 ± 0.03	144.96 ± 7.71
3	10.68 ± 0.23	23.66 ± 0.55	25.2 ± 1.69	3.39 ± 0.18	1.66 ± 0.03	140.53 ± 7.00
4	18.13 ± 7.97	34.00 ± 10.84	28.4 ± 1.34	3.75 ± 0.24	1.68 ± 0.09	138.56 ± 7.85
5	18.10 ± 10.80	35.31 ± 15.97	28.8 ± 3.50	3.98 ± 0.97	1.71 ± 0.07	126.37 ± 8.77

## Data Availability

The data will be provided upon reasonable request.
